# Metal Contents in Water, Sediment, and Oligochaeta-Chironomidae of Lake Uluabat, a Ramsar Site of Turkey*

**DOI:** 10.1100/tsw.2010.117

**Published:** 2010-07-06

**Authors:** Naime Arslan, Burcu Koç, Arzu Çiçek

**Affiliations:** ^1^ Biology Department, Science and Art Faculty, Eskişehir Osmangazi University, Eşkisehir, Turkey; ^2^ Applied Research Centre for Environmental Problems, Anadolu University, Eskişehir, Turkey

**Keywords:** metal, Lake Uluabat, zoobenthos, Oligochaeta, Chironomidae

## Abstract

Samples of lake water and sediment, and sediment and two dominant zoobenthic taxa (Oligochaeta: *Potamothrix hammoniensis* and Chironomidae: *Chironomus* [*Camptochironomus*] tentans larvae), were collected from 12 stations in Lake Uluabat and examined from the metal level point of view (cadmium, chromium, lead, copper, nickel, and zinc). Our results showed that the occurence of metals in water, sediment, and the two zoobenthic taxa are relatively high. The opinion that supports the results of Lake Uluabat shows that certain species of oligochaetes and chironomids accumulate examined metals several times over compared to their surroundings. Therefore, it is concluded that the oligochaetes and the chironomids are suitable candidates to be used in biomonitoring surveys of Lake Uluabat.

## INTRODUCTION

During the last years, not only industrial activities, but also anthropogenic activities have had negative consequences for the natural environment. This unfavorable impact has also been seen in aquatic ecosystems. All aquatic organisms accumulate trace metals in their bodies whether or not these metals are essential to metabolism. It is well known that metals accumulate in tissues of aquatic animals and, therefore, the levels measured in tissues of aquatic animals can reflect the past exposure. With the exception of fish tissues and organs that are frequently used as bioindicators of metal pollution, invertebrates are used to a lesser extent, although it has been shown that they can provide useful information on the pollution status of a particular area[1]. Bioaccumulation of toxic metals by benthic fauna also requires attention because of the potential for transferring contaminants up the food web to human consumers[2]. Benthic invertebrates are often the main diet for many predators, especially for fish,

*This study is a part of the Ph.D. thesis of Burcu KOÇ, supervised by Naime Arslan and Arzu Çiçek.
**Corresponding author.
©2010 with author.
Published by TheScientificWorld; www.thescientificworld.com and may remarkably contribute to the transfer of metals to higher trophic levels. Although determinations of trace metals in benthic invertebrates started in the last few decades in Turkey, our knowledge of metal concentrations in different benthic species is still sparse. In the present study, some detritus feeders (oligochaetes, chironomids) were used because they are closely associated with surficial sediments, burrowing in the upper layer and feeding on particulate matter, and these two benthic invertebrates are an important food source for fish[3,4]. Therefore, these two animal groups can be an important vector for the movement of chemicals out of sediment deposits and into the water column and terrestrial food chains. In addition, oligochaetes and chironomid~~s~~ species are widely used in many toxicity tests, and they are increasingly used as environmental bioindicators, especially in polluted environments. However, few data are available on metal concentrations in natural communities of benthic invertebrates, especially from freshwater systems in Turkey. In the present study, chironomid larvae and oligochaetes were selected in order to study metal bioaccumulation by macroinvertebrates. As reported previously, they are dominant groups in the benthic fauna in Lake Uluabat[5]. It is known that these two benthic groups are tolerant to some levels of metals, their large size allows the analysis of individual organisms, and they are important in the ecological food chain. Both species are sediment dwellers and live in close contact with the sediment[6].

Lake Uluabat (also known as Lake Apolyont) is considered to be one of the most Important Bird Areas (IBA), not only in Turkey, but also in the Palearctic region[7]. In this respect, although important studies have been carried out in the last decades[8,9,10], very few studies have been reported on the accumulation of metals in both biotic and abiotic components of Lake Uluabat[11,12].

In the present study, concentrations of cadmium, chromium, lead, copper, nickel, and zinc were determined in lake water, bottom sediment, and two dominant macrozoobenthic taxa (Oligochaeta and Chironomidae larvae) for the following purpose:

1. To determine the metal content in the tissues of dominant species of the benthos
2. To establish the relationship between metal concentrations in Oligochaeta, Chironomidae, and concentrations in the water and in the bottom sediment
3. To determine cadmium, chromium, lead, copper, nickel, and zinc contents of Oligochaeta and Chironomidae samples as food for fish
4. To determine whether macroinvertebrates could serve as a bioindicator of metals

## MATERIAL AND METHODS

The concentration of some metals (Cd, Cr, Pb, Cu, Ni, and Zn) were examined monthly in lake water (for all samples at each sampling station, n = 9), sediment (n = 9), and dominant macrozoobenthic samples (Oligochaeta, n = 9; Chironomidae larvae, n = 9) between August 2004 and July 2005 from 12 sites within Lake Uluabat.

### Study Area

Lake Uluabat has a tectonic origin and it is located between 44$^{o}$ 40' and 44$^{o}$ 60' N latitude and 62$^{o}$ 00' and 65$^{o}$ 00' E longitude in Bursa, Turkey, lying east to west, south of the Marmara Sea (Fig. 1). Lake Uluabat is one of the most important wetlands of Turkey. The lake is protected by the Ramsar Convention, 1998[7] and is currently considered as showing a typical eutrophication character.

It is a large, but shallow (maximally 3 m deep) freshwater lake, which covers an area of between 135 and 160 km$^2$, depending on the water level. A large and expanding delta has been formed by silt deposition around the Mustafakemalpaşa River mouth in the southwest section and its only outlet is in the northwest where it drains into the Kocaçay River. Most of the catchment area has been used for arable farming and willow and fruit plantations.

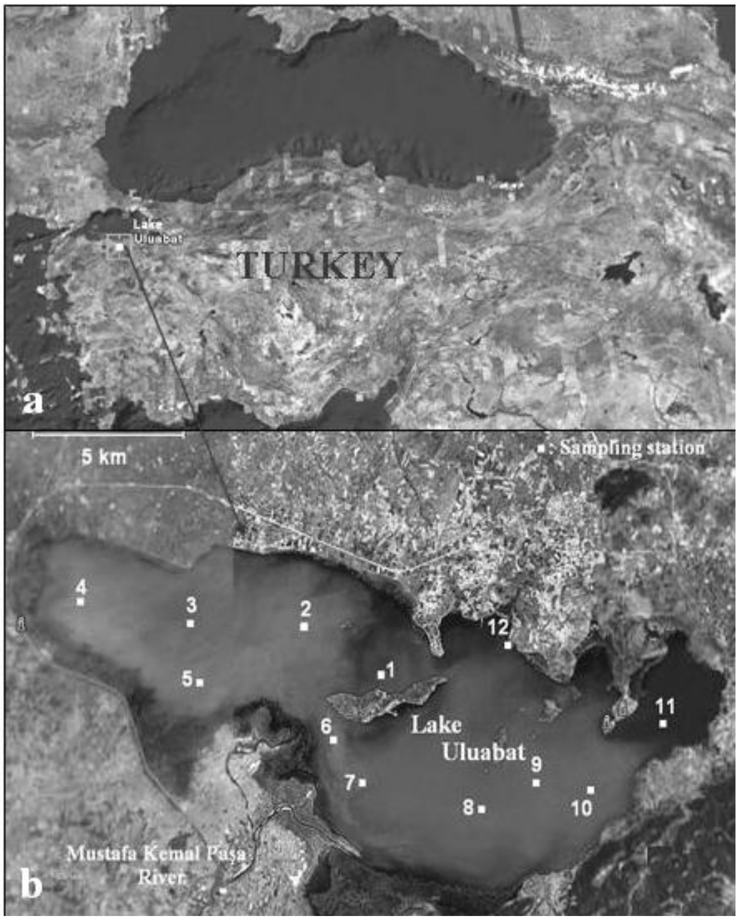

**FIGURE 1.** (a) Geographical situation of Lake Uluabat, Turkey; (b) sampling stations; (c) catchment area and the some emissions affecting the lake (next page).

Agricultural land and industries surround the lake, which is one of the most productive agricultural areas in Turkey due to its suitable climatic conditions, high-quality soils, and developed irrigation. Dalkıran et al.[10] indicated that the Lake Uluabat catchment is in one of the most productive agricultural regions of Turkey, with approximately 16 urban settlements on the lake shores. The main human activity at the lake is fishing. Besides fisheries and agriculture, stockbreeding is also an important activity around the lake[7].

In addition, up to 1985, crayfish (*Astacus leptodactylus* Eschscholtz, 1823) was the most important fishery product in Lake Uluabat. After 1985, crayfish production was reduced dramatically in Lake Uluabat as a result of the crayfish plague fungus (*Aphanomyces astaci*) whose presence was reported by several authors[13].

## Sample Collection

Two replicate bottom sediment, zoobenthic, and water samples were collected every month from 12 sites from Lake Uluabat from August 2004 to July 2005. However, because of bad weather conditions in December 2004 and in January-February 2005, samples could not be taken.

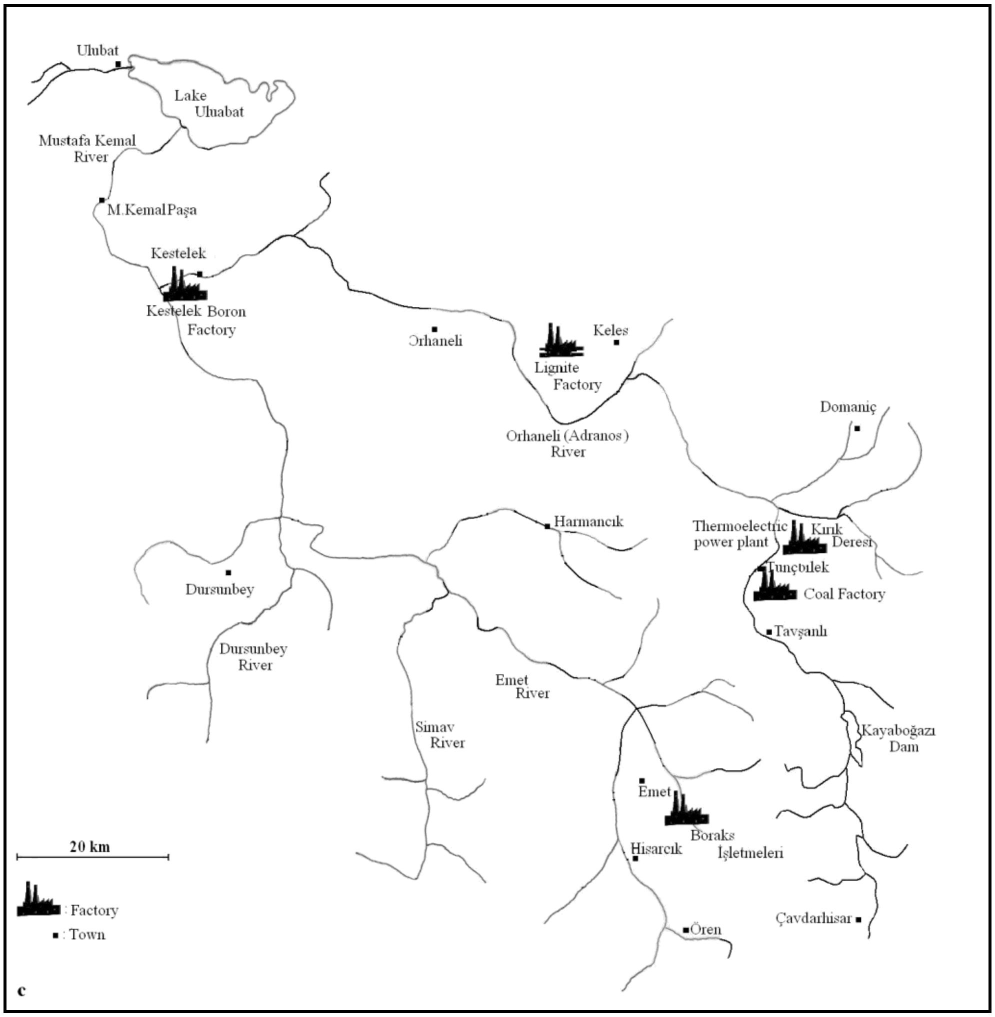

**FIGURE 1c**

## Water

Water pH, dissolved oxygen, and temperature were measured in the field with a DOK-TAO mark portable water quality checker (WQC-22A). Minimum, maximum, and average values of the environmental parameters determined in the lake water are given in Table 1. At the same time, water was collected in plastic bottles and kept cool for metal analysis in the laboratory. Water samples of 1 L in volume were taken at each sampling point and were adjusted to pH 2 by adding $HNO_3$. Before sampling, sample bottles were cleaned by washing them with detergent and then soaking them in 50% HCl for 24 h. Finally, the bottles were washed with distilled water. Bottles were kept in 1% nitric acid before their use.

## Sediment

At each sampling station, sediment was collected from three random sites and mixed. The upper layers of sediment were collected for metal analysis at all stations by using an Ekman-dredge (surface area 225 $cm^2$), taking small portions from the center of the dredge with a polyethylene spoon to avoid contamination by metallic parts of the dredge.

**TABLE 1**
**Sampling Station Coordinates, Median Basic Limnological Parameters***

| Ss | Coordinates | Parameters | | | |
|---|---|---|---|---|---|
| | | D | T | pH | BOD |
| 1 | 40º 10' 45 N<br>28º 35' 42 E | 1 | 20.4 (12–27.3) | 8.4 (8.2–9) | 13.8 (6–36) |
| 2 | 40º 12' 02 N<br>28º 33' 51 E | 2.1 | 19.9 (12.3–25.2) | 8.4 (8.1–8.9) | 7.6 (3–16) |
| 3 | 40º 11' 55 N<br>28º 31' 09E | 1.7 | 19.5 (11.5–24.8) | 8.4 (7.9–8.9) | 7.5 (2–17) |
| 4 | 40º 12' 08 N<br>28º 28' 26 E | 1.8 | 19.4 (11.2–24.5) | 8.3 (7.8–9) | 8 (2–27) |
| 5 | 40º 11' 08 N<br>28º 30' 15 E | 1.2 | 19.7 (11.8–24.7) | 8.4 (8.1–8.9) | 7.8 (2–16) |
| 6 | 40º 09' 58 N<br>28º 34' 10 E | 1 | 19.6 (11.1–25.6) | 8.3 (8–8.6) | 7.3 (1–15) |
| 7 | 40º 08' 20 N<br>28º 35' 11 E | 1.2 | 20 (10.9–25.4) | 8.3 (7.8–8.6) | 8.1 (1–18) |
| 8 | 40º 08' 09 N<br>28º 38' 43 E | 1.8 | 19.7 (11.3–26.7) | 8.4 (8.2–8.8) | 8.8 (2–23) |
| 9 | 40º 09' 26 N<br>28º 38' 31 E | 1.4 | 20.9 (12.8–26.7) | 8.3 (8.1–9) | 15.3 (4–40) |
| 10 | 40º 08' 31 N<br>28º 40' 21 E | 1.2 | 20.3 (12–26.6) | 8.3 (8–9.1) | 12.1 (8–23) |
| 11 | 40º 09' 23 N<br>28º 42' 54 E | 1.6 | 21.5 (27.1–13.9) | 8.4 (8.2–9) | 16.1 (6–42) |
| 12 | 40º 11' 36 N<br>28º 38' 12 E | 0.6 | 18.8 (13–23.3) | 8.3 (7.9–8.9) | 6 (3–11) |
| | | | Water Quality Criteria[1] | | |
| | Class I | — | 25 | 6.5–8.5 | 8 |
| | Class II | — | 25 | 6.5–8.5 | 6 |
| | Class III | — | >30 | 6.0–9.0 | 3 |
| | Class IV | — | >30 | 6.0–9.0 | <3 |

*Note*: Number in parantheses indicate min.-max. values; Ss: sampling station; D: depth (m); T: temperature (oC); BOD: biological oxygen demand (mg L–1).

[1] Turkish Environmental Guidelines[20].

## *Zoobenthos*

In each station, two seperate sediment samples were taken by using an Ekman-dredge for evaluation of community structure and for metal analysis. The first sediment samples were washed *in situ* using a 200-μm mesh size. They were preserved in 4% formalin in the field for later identification in the laboratory of the zoobenthic community structure. All samples were identified to order, family, or genera levels. The second sediment samples were washed with lake water several times. Sediment was placed in petri dishes and examined, by parts, for the presence of oligochaetes and chironomid larvae. Twenty to 150 specimens of each group were collected directly by hand from the substratum, and then put into lake water and transported to the laboratory alive in cold conditions in plastic vials.

In the laboratory, in order to study the metal tissue content of oligochaetes and chironomids, one section of this experiment involved purging the gut contents of live specimens for 12 or 16 h at room temperature before carrying out metal analyses. After 12 or 16 h, all samples were cleaned with distilled water and dried at 105°C for 24 h, and then decomposed with 5 mL conc. HNO and 0.5 mL $H_2O_2$, with following dilution to 25 mL. Whole oligochaetes and chironomid larvae were analyzed for metal concentrations. Species of the oligochaete *Potamothrix hammoniensis* (Family Tubificidae) and the midge *Chironomus* (*Camptochironomus*) *tentans* were chosen for accumulation measurements because of their wide abundance in the lake and their importance in the ecological food chain. Both species are sediment dwellers.

## Metal Analysis

Six metals were analyzed in the water, sediments, chironomid larvae, and oligochaetes: cadmium (Cd), chromium (Cr), lead (Pb), copper (Cu), nickel (Ni), and zinc (Zn).

After the gut contents of the oligochaete and chironomid specimens were purged, they were deep frozen at about –20°C and stored until analyses. Then, all chironomid and oligochaete samples were dried for 24 h at 105°C.

Air-dried bottom sediment samples were dried for 3 h at 105°C for metal analysis. After all samples had been passed through a nylon sieve (0.5 mm), 0.5 g of each sample was placed in pyrex reactors of a CEM Star 2 microwave digestion unit. $HClO_4$:$HNO_3$ acids of 1:3 proportions for samples were inserted in the reactors, respectively. Samples were mineralized at 200°C for 30 min. Afterwards, the samples were filtered in such a way as to make their volumes to 100 mL with ultrapure distilled water.

Metals were determined by the flame atomic absorption spectrophotometric (Varian Spectra A 250 Plus model) method[14,15,16,17]. The metal analyses in water, bottom sediment, and specimens were recorded as means triplicate measurements. In the FAAS analysis, the following wavelength lines were used: Cr 357.9 nm, Cd 228.8 nm, Cu 324.8 nm, Ni 232.0 nm, Pb 217.0/283.3 nm, and Zn 213.9 nm. The analytical process quality was also controlled by certified reference biological material of NCS DC73350 and standard reference material NIST-SRM 1573a. The analysis of these standard reference materials showed good accuracy, with the recovery rates of the metals between 90 and 107% (Table 2).

**TABLE 2**
**Analysis of the Biological Standard (NIST) and Certified (NCS) Reference Materials (mg kg$^{-1}$ Dry Matter)**

| Metal | NIST-SRM 1573a | | NCS DC 73350 | |
|---|---|---|---|---|
| | Certified | Analytical Value | Certified | Analytical Value |
| Cu | 4.70 ± 0.14 | 4.85 | 9.3 ± 1.0 | 10 |
| Ni | 1.5 ± 0.07 | 1.51 | 1.9 ± 0.3 | 1.85 |
| Cr | 1.99 ± 0.06 | 1.93 | 0.55 ± 0.07 | 0.50 |
| Pb | — | — | 1.5 ± 0.3 | 1.52 |
| Zn | 30.9 ± 0.7 | 31.8 | 37 ± 3 | 33.5 |
| Cd | 1.52 ± 0.04 | 1.49 | — | — |

### *Statistical Analysis*

We also also calculated two measures of biodiversity: Shannon-Wiener index (*H'*) and evenness (*J'*=H'/log [taxon number]). Correlations between metal concentrations in oligochaete and chironomid samples, and metal concentrations in water and sediments, were calculated by means of Spearman's correlation coefficients. All calculations were done with the program CSS Statistica 3.1 (StatSoft. Inc., 1993).

## RESULTS

### Zoobenthos Diversity and Structure of Lake Uluabat

The present study results show that oligochaetes were the most abundant group of animals in the lake benthos, comprising on average 35.6% of the total number of individuals. The phylum Nematoda constituted on average 27.7% of meiofaunal animals and the dipteran family Chironomidae larvae constituted on average 12.3% of macrofaunal individuals. Gastropods were the fourth most abundant group of animals in the lake, comprising on average 10.7% of the total number of individuals. Ostracods comprised 3.6% of meiofaunal individuals, and 10.1% were other animals (Bivalvia, Ceratopogonidae, Hirudinea, Odonata, Ephemeroptera, Asilidae, Hydracarina, Hemiptera, Argulidae, and Gammaridae). The highest biodiversity and high taxon richness was found at sampling station 12. The number of taxa and Shannon-Wiener index (1.68) in this station differed from the values found at all other stations. This flooded sampling site differed from the other sites with respect to depth, densely populated by macrophytes (dominated by *Nympha* spp.). This can be explained by the macrophytes increasing habitat heterogeneity, improving food conditions, and stabilizing bottom sediments[18].

The zoobenthic diversity of sampling sites 5 and 11, which were devoid of plants, was low. The lowest numbers of taxa (7–8) and lowest Shannon-Wiener indices (0.91–0.98) were found in these two sampling sites. Our results showed that the amount and diversity of zoobenthos in Lake Uluabat was not high. It was determined that oligochaete populations in the lake consisted mainly of *P. hammoniensis* (Michaelsen, 1901), *Tubifex tubifex* (Müller, 1774), and *Psammoryctides albicola* (Michaelsen, 1901). In addition, the most abundant chironomid species was *C.* (*Camptochironomus*) *tentans* (Fabricius, 1805), which showed the highest numbers at all sampling sites in the lake. Altough the second most abundant species was *Tanypus punctipennis* (Meigen, 1818) and the third was *Procladius choreus* (Meigen, 1804), they were not used in the metal analyses.

### Environmental Parameters in Water

All water samples showed high pH values generally >8, with total range between 7.8 and 9.1 (Table 1). The temperature of the sampling stations reflected the seasonal changes and ranged from 11.2 to 27.3°C.

Water quality criteria in Turkey divide inland waters into four classes. Quality level I refers to clean water that can be used for domestic purposes after simple disinfection, for recreational purposes, or for irrigation. Quality level II refers to fairly clean water that can be used as domestic water after treatment, for recreational purposes, or for fishing, farming, etc. Quality level III includes polluted water, which can only be used as industrial water after treatment. Class IV refers to heavily polluted water that should not be used at all. According to the Turkish water regulation, BOD was found at the second or third quality level (except station 12), which refers to moderately polluted water (Table 1).

## Metals in Water and Sediment

Concentrations of examined metals in the sediments, lake water, oligochaete and chironomid samples are shown in Table 3 and Fig. 2.

**TABLE 3**
**Average Concentration of Metals in Water, Sediment, Oligochaete and Chironomid Samples and Literature Data**

| | Average Concentration of Metals (min.-max value ± SD) | | | | | |
|---|---|---|---|---|---|---|
| | **Cd** | **Cr** | **Pb** | **Cu** | **Ni** | **Zn** |
| Water (mg $L^{-1}$) | | | | | | |
| Present study | 0.003 (n.d.–0.018) | 0.017 (n.d.–0.132) | 0.105 (n.d.–0.401) | 0.118 (0.007–0.337) | 0.056 (n.d.–0.245) | 0.284 (0.363–3.85) |
| Elmacı et al.[12] | 0.04 | 0.021 | 0.025 | 0.141 | 0.022 | 0.13 |
| Sediment (DW, mg $kg^{-1}$) | | | | | | |
| Present study | 0.699 (n.d.–7.4) | 57.9 (n.d.–132.7) | 110.7 (n.d.–372.5) | 119.2 (38.6–289.8) | 209.4 (75.6–303.8) | 171 (17.2–395.7) |
| Elmacı et al.[12] | 2 | 9 | 13 | 12 | 8 | 1 |
| Zoobenthos (DW, mg $kg^{-1}$) | | | | | | |
| Oligochaeta | 0.676 (0.82–4.75) | 51.09 (n.d.–153.2) | 54.62 (94–102.3) | 194.91 (111.7–292.7) | 23.67 (n.d.–83.46) | 452.87 (278.1–637.2) |
| Chironomidae | 3.13 (4.47–6.68) | 31.20 (n.d.–160.4) | 1.60 (n.d.–3.13) | 460.8 (133–985) | 93.04 (40–147) | 417.4 (179.4–759) |
| Water Quality Criteria (mg $L^{-1}$) | | | | | | |
| Turkish guidelines[1] | | | | | | |
| Class I | 0.003 | 0.02 | 0.01 | 0.020 | 0.02 | 0.2 |
| Class II | 0.005 | 0.05 | 0.02 | 0.050 | 0.05 | 0.5 |
| Class III | 0.01 | 0.2 | 0.05 | 0.2 | 0.2 | 2 |
| Class IV | >0.01 | >0.2 | >0.05 | >0.2 | >0.2 | >2 |
| EPA[2] | | | | | | |
| CMC | 0.004 | 0.016 | 0.065 | 0.013 | 0.470 | 0.120 |
| Sediment Quality Criteria[3] (mg $kg^{-1}$) | | | | | | |
| PEC | 4.98 | 111 | 128 | 149 | 48.6 | 459 |
| TEL | 0.596 | 37.3 | 35 | 35.7 | 18 | 123 |
| LEL | 0.6 | 26 | 31 | 16 | 16 | 120 |
| MET | 0.9 | 55 | 42 | 28 | 35 | 150 |
| TEL-HA28 | 0.58 | 36 | 37 | 28 | 20 | 98 |

*Note*: Number in parantheses indicated min.-max. values; DW: dry weight; n.d.: not detected.

1 Turkish Environmental Guidelines[20].

2 According to EPA[19], CMC: criteria maximum concentrations.

3 According to MacDonald et al.[25], PEC: probable effect concentrations; TEL: threshold effect level; LEL: lowest effect level; MET: minimal effect threshold; TEL-HA28: threshold effect level for *Hyalella azteca* (28-day test, DW).

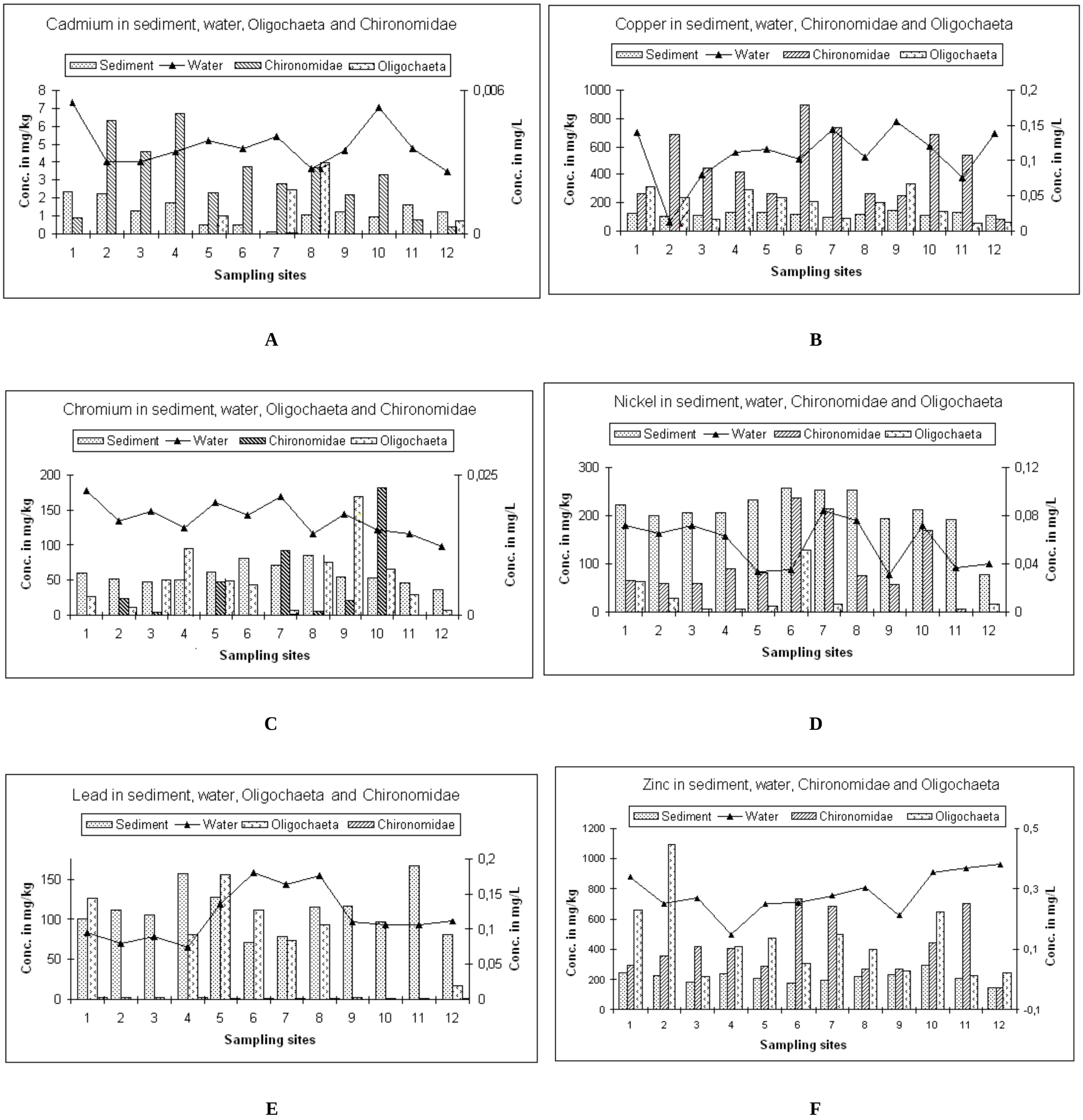

**FIGURE 2.** Concentrations of metals in the sediments, lake water, oligochaete and chironomid samples (for sediment, oligochaete, and chironomid samples mg $kg^{-1}$ dry matter).

It is known that some metals are essential for organisms (such as zinc, copper, and iron), but some of them are considered toxic to aquatic organisms, especially at higher concentrations (such as cadmium, lead, and chrome). Zinc is known as an essential metal and among the elements analyzed, it was found in the highest concentrations in the lake water at all sampling sites (except sites 5, 7, 8, and 9), while lead was found in the highest concentrations at sites 5, 7, and 8 (Fig. 2). These two metal concentrations in lake water were higher than the criteria maximum concentration (CMC) limits given by the EPA (0.120 and 0.065 mg $L^{-1}$, respectively)[19]. According to average values, the metal levels were as follows for the water in Lake Uluabat: Zn > Cu > Pb > Ni > Cr > Cd. According to the values established for water quality criteria in surface water by the Turkish Government[20], metal concentrations (especially lead, copper, and nickel) in the lake water surpass the upper limits (cf. Table 3). In water samples of Lake Uluabat, zinc and copper concentrations were higher. These results were in agreement with those obtained by Elmacı et al.[12] for the same sites.

The metal concentrations found in the upper layer of the sediments of the 12 sampling sites varied considerably. Except the essential metals (zinc and nickel), lead was found in the highest concentrations at three sampling sites (3, 5, and 11) (Fig. 2).

The results could be explained by the fact that cadmium and chromium in sediments are mainly associated with the carbonate fraction and are readily solubilized metals. Domestic and industrial effluents, municipal runoffs, and atmospheric deposition may be the major sources of the observed high levels of zinc, nickel, copper, and lead.

## Metals in Oligochaetes and Chironomid Larvae

The metal concentrations in the oligochaetes and chironomid larvae are shown in Table 3 and Fig. 2. Wide variation was shown in metal concentrations between oligochaete and chironomid samples (Table 3). Our results showed that zinc concentration was highest (average 452 mg $kg^{-1}$) in oligochaetes, while copper concentration was highest (average 460.8 mg $kg^{-1}$) in chironomids. Chironomids had lower lead concentrations than oligochaetes, but the cadmium, chromium, and zinc concentrations did not clearly vary between the two invertebrate groups. Our results show that concentrations of lead in oligochaetes are about 55 times higher than in the chironomid larvae, while concentrations of copper in chironomid larvae are about two to three times higher than in oligochaetes.

High metal concentrations in Oligochaeta and Chironomidae samples indicate metal entrance through the food chain. The accumulation of the metal in Oligochaeta and Chironomidae depends on several factors, such as absorption properties of the species. Our results show that examined invertebrates accumulate all elements between 10 and 100 times when compared to their surroundings.

## Statistical Analysis

The zoobenthic community diversity was evaluated according to the Shannon-Wiener index. According to this index, the zoobenthic community diversity in Lake Uluabat was found to be 1.32 on average; stations 11 and 12 had the highest diversity (1.68 and 1.64, respectively), while stations 5 and 11 had the lowest diversity (0.91 and 0.97, respectively).

Metal levels in the oligochaetes and chironomid larvae were related to metals in water and sediment. Significant and positive Pearson correlation coefficients were found between chromium in water and cadmium in Chironomidae ($p < 0.01$, r = 0.323). In addition, chromium and cadmium in sediment showed significant positive correlation coefficients with chromium and cadmium in Chironomidae, respectively ($p < 0.01$, r = 0.409; r = 0.383). Lead and cadmium in sediment showed a significant positive correlation with lead and cadmium in Oligochaeta, respectively ($p < 0.01$, r = 0.465; $p < 0.05$, r = 0.215). In the present study, we found significant negative correlations between levels of cadmium and lead in sediment and copper and chromium in Chironomidae samples, respectively ($p < 0.01$, r = –0.318; r = –0.231).

## DISCUSSION AND CONCLUSIONS

Certain species of oligochaetes and chironomids are widely used in many toxicity tests, but in Turkey, few data are available on metal concentrations in natural communities of them, especially from freshwater sources. Zoobenthic forms, such as oligochaetes and chironomids, are often the main diet for many fish species (especially Cyprinids) and may contribute to the transfer of metals to higher trophic levels.

Hellawell[21] reported that metal toxicity is affected by temperature, dissolved oxygen concentrations, and pH. Cadmium is very toxic to fish and other aquatic organisms; however, temperature, pH, and water hardness are factors that influence its toxicity and uptake by fish[11]. In Lake Uluabat, pH values were high and other parameters varied within the normal range. It is known that lake water quality is poor due to sewage water, agricultural fertilizers and chemicals, animal waste, processed wastes of food-stuff industries, tannery wastes, slaughterhouse wastes, and mining wastes from the watershed and pesticides from agricultural runoff[9,11,22]. Our results support the previous data. According to our results, the metals lead, copper, nickel, and zinc had levels higher than the Turkish Environmental Guidelines[20] limits in water. In addition, concentrations of all the metals are at least 100–200 times higher in the sediments than in the lake water. Lake sediment represents an important sink for trace metals in aquatic systems, and metal concentrations in sediment can be several orders of magnitude greater than in the overlying water. Sediment-associated metals pose a direct risk to detrital and deposit-feeding benthic organisms, and may also represent long-term sources of contamination to higher tropic levels[23,24]**.** Fish living in the polluted waters may accumulate toxic trace metals via ingestion of benthic organisms with elevated metal levels. As we mentioned above, high metal concentrations in oligochaetes and chironomids may imply that metals might be transferred up the food chain. The probable effect concentrations (PEC, Table 3) for sediment levels were reported by MacDonald et al.[25]. According to the results of chemical analysis in sediment samples (Table 3), the mean concentrations of copper and lead in sediment are much higher than the probable effect concentrations reported by MacDonald et al.[25]. In addition, according to the highest concentrations, of all metals surpass the PEC. As we mentioned above, the zoobenthic faunal composition of Lake Uluabat was not diverse. It is known that the lake receives untreated industrial, municipal, and a variety of agricultural wastewaters and past activities are also important for metal contamination of the lake. Dalkıran et al.[10] indicated that the Uluabat catchment is in one of the most productive agricultural regions of Turkey, with approximately 16 urban settlements on the lake shores. In addition, they emphasized that a high pollution load, entering Lake Uluabat via the Mustafakemalpaşa River, is one of the most important reasons for the external pollution load. Furthermore, Elmacı et al.[12] reported that the pollution that occurs from the Mustafakemalpaşa River and its tributaries (Orhaneli and Emet Streams) is directly transported to the lake. A part of the concentrated wastewaters of the active chrome mine of Harmancık at Emet Stream is discharged through the Kınık Stream. Also it reaches to the Emet Stream through the Kınık Stream after discharging. Our results showed that lead, chromium, and cadmium concentration levels in sediment samples were about eight, six, and three times higher, respectively (Table 3), than reported by Elmacı et al.[12]. However, data obtained from other studies suggest that inflow from the Mustafakemalpaşa River is the major source of both nutrients and accelerated siltation[11,26,27]. Lammens and van den Berg[26] report fluctuating nutrient levels linked to changing river input and reduced internal plant macrophyte cover compared to a decade earlier.

Although knowledge of metal concentrations in different benthic species in Turkey is sparse, oligochaetes and chironomid larvae are widely used in ecotoxicological studies. In a similiar study, conducted by Tulonen et al.[28], the concentrations of metals in different food web components, such as zooplankton, benthic invertebrates, and fish from southern Finland, were shown. They showed that in some detritus feeders (oligochaetes, chironomids), metal concentrations were higher than in other invertebrate groups, which may also indicate the presence of species-specific uptake mechanisms for different metal ions. In addition, a recent surge in publications related to dietary metal accumulation in a variety of organisms[29,30,31] confirms earlier work[32] that diet can be a major source of metal accumulation in aquatic organisms. Fish, especially carnivorous fish, are often at the top of the aquatic food chain and may accumulate large amounts of some metals via food chain. Accumulation patterns of contaminants in fish depend both on accumulation rate and feeding. It is reported that 21 fish species are living in Lake Uluabat[33]. Among them, three species, *Esox lucius*, *Carasssius gibelio,* and *Scardinus erythrophthalmus,* are important sources of protein for human nutrition in the region. Therefore, the presence of metals in fish from Lake Uluabat possesses a risk for food contamination. Although some metals, such as iron, copper, zinc, and manganese, are essential metals since they play important roles in biological systems, some metals, such as lead and cadmium, are toxic, even in trace amounts.

Metal incorporation by aquatic organisms may occur through active ingestion or by a passive uptake via external body surfaces[34], and can come from different sources. It is reported that correlations between metal concentration in aquatic organisms and their environment are often weak because bioaccumulation depends on many factors, including both the individual (age, size, or nutrition state) and the environment (metal speciation or chemical conditions)[35]. However, correlations were found between chironomid larvae-oligochaetes and in water sediment, indicating a strong uptake of these metals from the water and (or) sediment.

From our results, it is clear that the concentration of metals in the examined compartments of Lake Uluabat is high. Hence, we conclude there should be an effort to protect Lake Uluabat from pollution to reduce environmental risk. In addition, this study may provide valuable data for future research on Lake Uluabat. The main topics that may need to be investigated are control of all discharges, regular observation of pollutants, and evaluation of the effect of pollutants on the lake's ecosystem over the long term.

## ACKNOWLEDGMENT

This work was partly supported by grants from the Scientific Research Projects Fund of Ekişehir Osmangazi University (project no. 200419007), Turkey. The authors are grateful to Dr. Tugrul Onturk, Seval Kökmen, Dr. Özgür Emiroğlu, and Dr. Semra Malkoç, who kindly provided a collection of samples and analyses for the study, and we also thank the fisherman, Aydogan Uysal, and his family for their help.
